# Supplementary material for: Retention and predictors of attrition among patients who started antiretroviral therapy in Zimbabwe’s national antiretroviral therapy programme between 2012 and 2015
Source: PLoS One. 2020 Jan 7;15(1):e0222309. doi: 10.1371/journal.pone.0222309 (PMC6946589; doi:10.1371/journal.pone.0222309)
Supplement: S1 File — (PDF) [file pone.0222309.s001.pdf]

Patient Retention, Clinical Outcomes and Attrition-Associated Factors of HIV-Infected Patients Enrolled in Zimbabwe's National Antiretroviral Therapy Programme, 2007–2010

Tsitsi Mutasa-Apollo[1]*, Ray W. Shiraishi[3], Kudakwashe C. Takarinda[1], Janet Dzangare[1], Owen Mugurungi[1], Joseph Murungu[1], Abu Abdul-Quader[3], Celia J. I. Woodfill[2]

1 AIDS and TB Department, Zimbabwe Ministry of Health and Child Care, Harare, Zimbabwe, **2** Division of Global HIV/AIDS, Centers for Disease Control and Prevention (CDC), Accra, Ghana, **3** Division of Global HIV/AIDS, Center for Global Health, Centers for Disease Control and Prevention (CDC), Atlanta, Georgia, United States of America

Abstract

Background: Since establishment of Zimbabwe's National Antiretroviral Therapy (ART) Programme in 2004, ART provision has expanded from <5,000 to 369,431 adults by 2011. However, patient outcomes are unexplored.

Objective: To determine improvement in health status, retention and factors associated with attrition among HIV-infected patients on ART.

Methods: A retrospective review of abstracted patient records of adults ≥15 years who initiated ART from 2007 to 2009 was done. Frequencies and medians were calculated for rates of retention in care and changes in key health status outcomes at 6, 12, 24 and 36 months respectively. Cox proportional hazards models were used to determine factors associated with attrition.

Results: Of the 3,919 patients, 64% were female, 86% were either WHO clinical stage III or IV. Rates of patient retention at 6, 12, 24 and 36 months were 90.7%, 78.1%, 68.8% and 64.4%, respectively. After ART initiation, median weight gains at 6, 12, and 24 months were 3, 4.5, and 5.0 kgs whilst median CD4+ cell count gains at 6, 12 and 24 months were 122, 157 and 279 cells/µL respectively. Factors associated with an increased risk of attrition included male gender (*AHR* 1.2; 95% CI, 1.1–1.4), baseline WHO stage IV (*AHR* 1.7; 95% CI, 1.1–2.6), lower baseline body weight (*AHR* 2.0; 95% CI, 1.4–2. 8) and accessing care from higher level healthcare facilities (AHR 3.5; 95% 1.1–11.2).

Conclusions: Our findings with regard to retention as well as clinical and immunological improvements following uptake of ART, are similar to what has been found in other settings. Factors influencing attrition also mirror those found in other parts of sub-Saharan Africa. These findings suggest the need to strengthen earlier diagnosis and treatment to further improve treatment outcomes. Whilst decentralisation improves ART coverage it should be coupled with strategies aimed at improving patient retention.

Citation: Mutasa-Apollo T, Shiraishi RW, Takarinda KC, Dzangare J, Mugurungi O, et al. (2014) Patient Retention, Clinical Outcomes and Attrition-Associated Factors of HIV-Infected Patients Enrolled in Zimbabwe's National Antiretroviral Therapy Programme, 2007–2010. PLoS ONE 9(1): e86305. doi:10.1371/journal.pone.0086305

Editor: Matthew P. Fox, Boston University, United States of America

Received June 28, 2013; **Accepted** December 8, 2013; **Published** January 29, 2014

This is an open-access article, free of all copyright, and may be freely reproduced, distributed, transmitted, modified, built upon, or otherwise used by anyone for any lawful purpose. The work is made available under the Creative Commons CC0 public domain dedication.

Funding: The survey was supported by the President's Emergency Plan for AIDS Relief (PEPFAR) through the U.S. Centers for Disease Control and also the Expanded Support Program for HIV/AIDS (ESP). The funders had no role in the study design, data collection and analysis, decision to publish, or preparation of the manuscript.

Competing Interests: The authors have declared that no competing interests exist.

* E-mail: tsitsiapollo1@gmail.com

Introduction

Globally, remarkable progress has been made in improving access to ARVs whereby treatment coverage of 65% (9.7 million people) [1] was achieved by end of 2012 compared to the 2015 target of 15 million agreed by United Nations Member States in June 2011 [2]. Of all these HIV-infected people already on ART, 16% (1.6million people) were put on ART in 2012 alone [1]. In Sub-Saharan Africa (SSA) which constitutes 69% of HIV infections globally, access to ART increased from 50,000 to more than 7.5 million between 2002 and 2012 [1].

Whilst there is need to scale up provision of this life-saving treatment, there is need to monitor and ensure retention in care of this growing cohort of patients so as to ensure ongoing receipt of ART, evaluate the emergence of medication toxicities, and identify the occurrence of treatment failure in order to switch regimens [3]. The high attrition occurring among HIV-infected patients in ART care in SSA has been widely documented, with a meta-analysis of 39 cohorts from SSA showing that patient

retention declined from 86% at 6 months to 77% by 36 months after ART initiation. [4] In West Africa patient retention has been reported at 76% by 1-year of follow-up whilst in Southern Africa 1-year retention ranges from 67–70% in Mozambique [5] to 82.7% in Botswana [6]

To further accelerate ART service coverage in SSA, there have been recommendations to scale-up decentralisation of ART services to primary health-care facilities coupled with task-shifting of service provision to nurses [7]. This has resulted in increased ART coverage in Malawi [8] and improved adherence and retention [9,10,11].

Zimbabwe, with a population of 12,9 million is among countries in the sub-Saharan region experiencing a mature HIV epidemic with a decline in prevalence over the last decade from 27.2% in 1998 to 14.0 in 2012 whilst HIV incidence stabilised at 1.0% in 2012 [12]. The adult HIV prevalence observed in the 2010–2011 Zimbabwe Demographic and Health Survey showed slightly higher HIV prevalence in urban areas than in rural areas and ranged by province from 13% in Harare to 21% in Matebeleland South [13]. The number of people living with HIV in 2012 was estimated to be 1.3 million whilst the total estimated number of people in need of antiretroviral therapy (ART) in 2012 was 621,673 adults and 108,263 children (based on a CD4<350 cells/mm3) [12].

Zimbabwe suffered a socioeconomic crisis between 2001 and 2009 with extreme recession and hyperinflation which significantly affected the national health system. During the crisis, there were extreme shortages of health workers and essential medical supplies [14]. The National Opportunistic Infections/Antiretroviral Therapy (OI/ART) Programme in Zimbabwe was established in April 2004. Since 2008, both ART initiation and follow-up services have been decentralised to lower level health facilities. In the period under review in this study, there was a remarkable increase in number of health facilities offering ART services and number of patients receiving treatment; by January 2007 there were 101 sites [15] providing treatment to 66,920 patients (11% coverage) [16] which rose to 337 sites [17] offering ART to 218,589 patients (49% coverage) [18] by December 2009 based on the WHO 2006 Guidelines.

The rapid expansion of ART programmes in settings with weak health systems is associated with high mortality and loss of patients to follow up [19,20]. With the on-going rapid expansion and decentralization of ART in Zimbabwe; it was not clear how treatment outcomes compared across levels of care. The purpose of this study was therefore to i) describe baseline demographic and clinical characteristics, and to determine ii) patient retention in care iii) clinical outcomes and iv) factors associated with attrition among HIV-infected patients enrolled in the National ART Programme in Zimbabwe.

Methods

Study Design, Study Population and Sampling

A nationally representative multi-stage retrospective cohort study was implemented using routine data from standard Ministry of Health and Child Care (MOHCC) ART monitoring tools (e.g., facility patient medical records). Between October and December 2010, trained MOHCC personnel abstracted patient-level data from facility medical records onto study questionnaires. Adult patients ≥15 years old at ART initiation who initiated ART between 2007 and 2009 at selected sites, and at least 12 months prior to date of chart abstraction were eligible to be included in the assessment (note: transfer-ins were excluded from the site-level sampling frame).

To achieve a 95% confidence interval (CI) of +/−2.5% around the estimate for a conservative 12-month patient retention of 50%, assuming a design effect of 2 and that 20% of medical records were missing, it was determined that a sample size of ≥3,684 patients was needed. In order to simplify the sampling strategy, we aimed to sample 4,000 charts of HIV-infected adults who initiated ART at least 12 months prior to the date of chart abstraction.

During study planning, MOHCC reported data from May 2008 were used to define the clinic sample frame. By 31 May 2008, 103,806 adults had initiated ART at 104 public sector sites and 70 of the sites had ≥50 adult ART patients and hence they were eligible for inclusion in the sampling frame. Sites were stratified by site size (Small: 50–249 patients; Medium: 250–1000 patients; Large: >1000 patients) and 40 facilities were selected via stratified probability -proportional-to-size (PPS) sampling. Patient charts were randomly selected at the 40 sites and the number of charts selected was proportional to the size of the site. Patient charts selected for data abstraction were searched for and retrieved by clinic staff. Missing patient charts were replaced by the next eligible patient record on the list of selected charts until the sample size for the site had been reached.

Data from medical records were abstracted onto paper-based questionnaires by trained data abstractors. For quality control purposes, supervisors re-abstracted 10% of all abstracted medical records. Data from the paper questionnaires were then hand entered into an MS Access database and separately scanned into a Teleform database after the data abstraction was completed. Data from the two databases were compared using the COMPARWS Macro [21] in SAS 9.2 (SAS Institute Inc., Cary, NC) to identify discrepancies. Discrepancies were then resolved by referring back to the paper questionnaires before the final dataset was cleaned prior to data analysis.

Ethics review

This study was approved by the Medical Research Council of Zimbabwe (MRCZ) and by the Institutional Review Board (IRB) of the United States Centers for Disease Control and Prevention. Both institutional review boards waived the need for written informed consent from the participants since data in this study were abstracted from existing medical records.

National ART Guidelines

During 2007–2009, adults were eligible for ART if they were HIV positive and had a diagnosis of World Health Organization (WHO) clinical stage IV, irrespective of CD4+ count; WHO clinical stage III and a CD4+ cell count <350 cells/μL; or WHO clinical stage I or II and a CD4+ cell count <200 cells/μL. Co-trimoxazole preventive therapy (CPT) was indicated for all symptomatic HIV-infected patients (clinical stages II, III and IV) and/or if CD4+ cell count was <200 cells/μL. Psychosocial criteria for ART initiation, included completion of prescribed counselling sessions and an assessment of adherence to CPT in the past 3 months. Adherence to CPT was used to assess the likelihood that the patient would adhere to ART.

According to national ART guidelines CPT adherence is assessed by health workers through pill counts and self-reporting by patients during each clinic visit and then recorded in the health facility held card.

Recommended first-line ART regimens included Stavudine, Lamivudine and Nevirapine. Single drug substitutions within the same drug class were recommended for serious drug toxicities and tuberculosis e.g. Zidovudine was used as an alternate 1st line for Stavudine while Efavirenz was substituted for Nevirapine. After initiating ART, a patient is seen every 2 weeks for the 1st month;

thereafter monthly for another 3 months. This is important as patients may present with inter-current illnesses, adverse drug events, or immune reconstitution syndrome. If a patient is stable after the initial 3 months; they are seen every 3 months thereafter. It is government's policy to provide ARV medicines free of charge to patients. However, patients are expected to pay consultation fees; laboratory-associated costs such as CD4 tests, Full Blood Count and radiography (CXR) for ruling out chest pathologies.

Recommended clinical and laboratory monitoring indices included patient weight, WHO clinical stage, development of opportunistic infections, complete blood count (CBC), Alanine Transaminase (ALT), serum creatinine, and where possible CD4+ cell counts. Routine clinical monitoring was to be conducted every 3 months; CBC and CD4 were to be conducted every 6 months; and ALT and creatinine were to be conducted every 12 months. CD4 testing was not widely available between 2007 and 2009 as it was mostly available in district hospitals or higher level facilities and at selected primary healthcare facilities which were initiating ART. In 2007 there were 46 CD4 machines and by 2009, 59 facilities in 47 out of the 62 districts countrywide had CD4 machines [22]. CD4 count testing was therefore done at each ART initiating sites if available, as there were no collection and transportation of blood samples to facilities with CD4 count machines. Patient demographic, clinical and laboratory information and visit dates are recorded in MOHCC medical records maintained at the health facility.

Treatment Outcome Measures

During data collection, the most recently documented status of each patient at the last clinic visit date was abstracted and recorded as alive-continued ART, transferred out, stopped, died, lost to follow-up (LFU), defaulted, and other. For this assessment, "retention" referred to a patient who was alive and known to be receiving ART at the same clinic where they initiated ART and "attrition" referred to a patient who was documented as having died, stopped ART, defaulted or was lost to follow-up. A patient defined as "defaulted" had been absent from a healthcare facility for ≤90 days whilst one who was "lost to follow-up" was absent for >90 days after his/her last scheduled appointment with the health care provider or pharmacy. The date of LFU was recorded as the date of the most recent visit or one day after ART initiation if patients only attended the initiation visit. Patient retention was calculated as the difference between date of ART initiation and the latest clinic visit date. Patients who were documented to have transferred out of a clinic before the time point of interest (either 6, 12, 24 or 36 months of follow-up) were excluded from the numerator and denominator of the relevant retention/attrition proportion.

For time-to-event analyses, patients who transferred to another clinic were censored at the date of transfer. However, for estimating retention proportions at 6, 12, 24, and 48 months, transfers were excluded from the retention analysis. Baseline CD4 was defined as the value on the date closest to ART start date but not more than 182 days prior to that date or more than 1 day after that date. Values for clinical stages, weights and CD4 counts at 6, 12 and 24 months of follow-up, were taken as values obtained on the dates closest to 182, 365 and 730 days, respectively.

Treatment failure was defined using clinical or immunological criteria. Viral load testing was not widely implemented due to the associated prohibitive costs. A patient on ART for at least 6 months presenting with new or recurrent WHO stage 4 condition (clinical failure) or a fall to CD4 count/percentage or persistent CD4 level below 100 cells (immunological failure) were considered treatment failure. The preferred 2nd line ART for adults that failed 1st line ART was Tenofovir, Lamivudine, and Lopinavir/Ritonavir.

Statistical Analysis

All analyses were performed using SAS 9.2 (SAS Institute Inc., Cary, NC) and Stata/IC 10.1 (StataCorp, 2009, Stata Statistical Software: Release 10.1, College Station, TX). Statistical analyses were weighted to account for unequal probabilities of selection and accounted for the complex design of the survey (i.e. stratification and clustering).

Missing data were assumed *missing at random* (MAR) [23] and were multiple imputed via chained equations using the ice [24–26] procedure in Stata. Twenty imputed datasets were created and estimates were combined according to Rubin's rules [23] using the mim procedure [27]. The imputation model included the Nelson-Aelen estimate of cumulative hazard [28], baseline demographic and clinical variables, and the event indicator. Time-to-event data were complete for all individuals. Twenty-five patients were lost to follow-up after their first clinic visit and were assigned one day of person-time.

Stratified Kaplan-Meier curves were used to graphically assess retention proportions. These analyses were limited to the first imputed dataset. Cox proportional hazards models were used to identify factors (e.g., year of ART initiation, gender, baseline CD4 count, baseline WHO stage, baseline body weight, active TB treatment, baseline haemoglobin) associated with attrition based on prior research. Hazard ratios (HRs), adjusted hazard ratios (AHRs) and their respective 95% confidence intervals (CI) and p-values were calculated. In multivariate-analysis HRs were adjusted for the potential confounding effects of sex, marital status, rural/urban location of health facility, baseline CD4 count, baseline WHO staging, baseline weight, prescribed CTX, level of health care and site size of ART clinic. The proportional hazards assumption was assessed via a variety of methods including log-log plots and Kaplan-Meier versus Cox predicted plots and results of these analyses suggested that the proportional hazards assumption appeared to be reasonable.

Results

Demographic and clinical characteristics of ART patients and characteristics of ART sites including clinical practices

A total of 3,919 medical charts of eligible ART patients were located and abstracted. Data were missing at baseline for 630 (16%) patients for WHO clinical stage, 2,085 (53%) for CD4 T-cell count, 1,049 (26.8%) for body weight, 3,021 (77%) patients for haemoglobin and 825 (21%) for cotrimoxazole prescription. **Table 1** describes analysis results for both the original and imputed datasets; in the following text, weighted imputed data are reported.

At enrolment into OI/ART, patient median age was 37 years, and 64% were female of whom 5.8% were pregnant. Fifty-two percent of the patients were married or cohabitating and 25.5% were widowed. Fifty-three percent of patients initiated treatment at an urban clinic and 46.7% initiated treatment at a rural clinic. Almost three quarters (72.6%) of patients initiated ART at a district or mission hospital; 16.8% initiated ART at a central or provincial hospital; and 10.6% initiated treatment at a primary healthcare facility.

Median CD4+ cell count at baseline was 121 cells/μL. Median CD4 cell count at baseline was slightly lower in males (104 cells/μL) than in females (127 cells/μL). At baseline, 22.4% of patients had a CD4 cell count less than 50 cells/μL, and 53.4% had a cell

Table 1. Demographic and clinical characteristics of patients at ART initiation and characteristics of ART sites, including clinical practices.

			All	Multiple Imputation (N = 3,919)
	N	N	Percentage (95% CI) Or Median (IQR)	Percentage (95% CI) Or Median (IQR)
Sex				
Female	2,514	3,907	64.1% (62.2–65.9)	64.0% (62.2–65.9)
Male	1,393	3,907	35.9% (34.1–37.8)	36.0% (34.1–37.8)
Missing	12	3,919	0.3%	–
Age At ART Start (Years)				
All	3,733	3,733	37 (32–45)	37 (32–45)
Missing	186	3,919	0.0%	–
Female	2,388	2,388	36 (31–44)	36 (31–44)
Male	1,335	1,335	39 (34–48)	39 (34–48)
Marital Status				
Single/Divorced	651	3,003	21.8% (18.1–25.4)	22.0% (19.0–25.2)
Married/Cohabiting	1,577	3,003	52.5% (49.8–55.2)	52.4% (50.1–54.7)
Widowed	775	3,003	25.7% (22.1–29.4)	25.5% (22.5–28.5)
Missing	916	3,919	23.4%	–
WHO Stage				
I/Ii	390	3,289	12.4% (6.8–18.1)	14.4% (8.6–20.1)
Iii	2,413	3,289	73.5% (66.9–80.1)	70.5% (63.9–77.1)
Iv	486	3,289	14.1% (9.7–18.4)	15.1% (11.4–19.0)
Missing	630	3,919	16.1%	–
CD4 T-Cell Count (Cells/Ml)				
≥200	712	1,834	22.7% (19.5–25.9)	24.1% (19.6–28.6)
50–<200	2,206	1,834	54.9% (51.2–58.7)	53.4% (49.8–57.1)
<50	1,875	1,834	22.4% (20.2–24.6)	22.4% (19.8–25.1)
Missing	2,085	3,919	53.2%	–
Weight				
>60 Kg	779	2,870	26.5% (22.9–30.1)	26.8% (23.5–30.0)
45–60 Kg	1,722	2,870	60.4% (57.9–62.3)	60.0% (57.6–62.3)
<45 Kg	369	2,870	13.1% (10.3–16.0)	13.3% (10.8–15.8)
Missing	1,049	3,919	26.8%	–
Prescribed CTX				
Yes	2,985	3,094	96.7% (95.0–98.3)	96.6% (95.2–98.1)
No	109	3,094	3.3% (1.7–5.0)	3.4% (1.9–4.8)
Missing	825	3,919	21.1%	
Site Type				
Rural	1,975	3,919	46.7% (22.0–71.3)	46.7% (22.0–71.4)
Urban	1,944	3,919	53.3% (28.7–78.0)	53.3% (28.6–78.0)
Missing	0	3,919	0.0%	–

doi:10.1371/journal.pone.0086305.t001

count between 50 and 200. A higher proportion of males had a CD4+ cell count below 50 cells/µL than females.

In terms of WHO clinical staging, 70.5% were diagnosed with stage III disease, 15.1% were diagnosed with stage IV disease, and the remaining were diagnosed with stages II and I disease. A slightly higher proportion of males were diagnosed with stage IV disease compared to females (males – 16.4% and females- 12.8%).

At baseline, 13.3% of patients weighed less than 45 kilograms (kgs) and 60% weighed between 45 and 60 kgs. The median weight of females was 53.2 kgs and of males was 57 kgs.

Treatment for tuberculosis at the time of enrolment into an OI clinic was documented for 12% of the 2,231 patients with information about TB treatment at OI clinic enrolment. A higher proportion of male ART patients were on TB treatment at OI clinic enrolment than female ART patients.

The majority of ART patients (96.6%) were prescribed CTX at baseline and 100% of the 3,759 patients with documented data were prescribed a first-line regimen. Three percent of patients had documented drug substitutions for active TB, toxicity, pregnancy and other reasons. Only six (0.2%) of patients had documented treatment failure and were switched to second-line antiretroviral treatment. Fifteen patients were documented to have stopped ART because of drug toxicity, pregnancy and other reasons.

Patient outcomes

Of 3,919 patients who initiated ART, 90.7%, 78.1%, 68.8% and 64.4% remained alive on ART at their initiating site after 6, 12, 24 and 36 months, respectively (**Table 2**). Retention on ART appeared higher in females when compared to males at 24 months (71.1% vs 64.6%) and at 36 months (66.9% vs. 59.7%).

Median follow-up duration was 1.36 years. By 6 months, 4.1% had died and 4.9% had been LFU; by 12 months, 4.8% had died and 16.1% had been LFU; by 24 months, 6.7% had died and 23.5% had been LFU; and by 36 months 8.5% had died and 25.2% were LFU. Of patients who died, 59.8% died within 90 days of starting ART. Of patients who were LFU, 15.6% were lost within 90 days and 68% were lost within 365 days of starting ART.

Clinical improvement was seen among both male and female patients. Increases in body weight and in CD4 cell counts were observed between baseline and follow-up appointments at 6, 12, and 24 months among patients with weight and/or CD4 cell count data. **Table 3** describes weight gains in ART patients.

Increases in CD4+ cells were also observed after ART initiation among the few patients with CD4+ count measurements (**Table 4**). For female patients, median CD4+ count gains from baseline were 122, 171, and 265 cells/µL at 6, 12, and 24 months, respectively. For male patients, median CD4+ count gains from baseline were 125, 130, and 179 cells/µL at 6, 12, and 24 months, respectively.

Factors associated with attrition

Table 5 shows the results of univariate and multivariable analyses of factors associated with attrition.

Male sex was associated with an increased risk of attrition (*AHR* 1.24; $p = 0.004$). Baseline WHO stage IV was associated with an increased risk of attrition (*AHR* 1.73; $p = 0.012$) as compared with WHO stages I/II. A baseline CD4 cell count<50 (*HR* 1.36, $p = 0.05$) was associated with higher attrition as compared with CD4 cell count >200 in univariate analysis, however, no associations between CD4 cell count and attrition were observed in the final multivariable survival model. Patients with baseline weights of 45–60 kilograms (*AHR* 1.25; $p = 0.02$) and <45 kilograms (*AHR* 2.03; $p = .000$) at baseline had an increased risk of attrition as compared with those patients with weights >60 kilograms (Figure 1).

While the majority of patients were prescribed cotrimoxazole prophylaxis at baseline, those who did not receive a cotrimoxazole prescription had significantly increased risk of attrition (*AHR* 1.94; $p = .007$).

Initiating ART at an urban site, as compared with a rural site, was marginally associated with attrition (*AHR* 2.13; $p = 0.06$). Initiating ART at a central/provincial hospital (*AHR* 3.21; $p = 0.06$) or at a district/mission hospital (*AHR* 3.49; $p = 0.04$) as compared with a primary healthcare clinic, was marginally associated with a higher risk of attrition (Figure 2).

Discussion

Clinical outcomes and retention in care were similar to findings in other Sub-Saharan African countries despite severe challenges to the health system, hence demonstrating that rapid ART scale-up with good clinical outcomes is feasible even under difficult circumstances. Retention rates in Zimbabwe are very similar to those estimated by Fox and Rosen [4] in a recent meta-analysis of published cohort data from 16 countries in sub-Saharan Africa in which estimated retention rates of ART patients at 6, 12, 24 and 36 months, were 86.1%, 80.2%, 70.0% and 64.6% respectively. Whilst our findings may have been similar to those in other Sub-Saharan African countries, we hypothesized that they would be worse. This is in view of existing hyperinflation in Zimbabwe during the period under review whereby cumulative inflation between 1997 and 2007 was 3.8 billion percent whilst the official inflation figure for February 2008 was 165,000 percent [29]. This hyperinflation was associated with increasing emigration of doctors and shortages of essential medical supplies in health facilities [14]. Despite this situation we managed to have comparatively similar findings through funding from bilateral aid and international multilateral institutions such as Global Fund to fight AIDS,

Tuberculosis and Malaria (GFTATM) and the Expanded Support Programme for HIV and AIDS (ESP) which ensured antiretroviral medicines were procured and distributed country-wide among other interventions. There was also support from ESP towards the human resource retention scheme [30] which supported primary counsellors who offered treatment adherence counselling and psychosocial support to patient whilst a significant amount was channelled towards retention of critical staff such as doctors who were responsible for initiating patients on ART, managing co-morbidities and complications as well as mentoring nurses in providing follow-up care. In 2009 alone, international sources contributed over 75% of total HIV spending [18] since internal funding sources such as the National AIDS levy were insignificant due to the eroded value of the Zimbabwe dollar.

Table 2. Retention of patients after 6, 12, 24, and 36 months.

		Total		Females		Males
	N	Percentage (95%CI)	N	Percentage (95%CI)	N	Percentage (95%CI)
6 months	3,739	90.7 (86.1–93.8)	2,413	91.9 (87.3–94.9)	1,316	88.5 (83.4–92.2)
12 months	3,641	78.1 (69.7–84.7)	2,352	79.6 (71.1–86.2)	1,280	75.3 (66.6–82.3)
24 months	2,003	68.8 (58.5–77.5)	1,286	71.1 (60.3–80.0)	711	64.6 (54.1–73.8)
36 months	806	64.4 (55.7–72.3)	522	66.9 (56.6–75.8)	282	59.7 (51.1–67.8)

Note: These analyses are not based on imputed data.
doi:10.1371/journal.pone.0086305.t002

Table 3. Median weight gains (kilograms) during ART stratified by gender.

	6-month gains		12-month gains		24-month gains		36-month gains	
	**Median (IQR)**	***N***	**Median (IQR)**	***N***	**Median (IQR)**	***N***	**Median (IQR)**	***N***
All	3.0 (−0.8–7.0)	1,621	4.5 (0.0–9.0)	1,444	5.0 (1.0–10.0)	665	6.0 (0.0–11.0)	209
Female	3.0 (−1.0–7.0)	1,058	5.0 (0.0–10.0)	955	5.0 (1.0–12.0)	429	6.0 (0.0–11.0)	134
Male	3.0 (0.0–6.8)	560	4.0 (0.0–8.0)	486	5.0 (1.0–9.8)	234	4.0 (0.5–9.0)	73

Note: These analyses are not based on imputed data.
doi:10.1371/journal.pone.0086305.t003

The median CD4 cell count at ART initiation was low although this was slightly higher than that reported in a systematic review of 39 patient cohorts in Sub-Saharan Africa [4]. We also note that nearly half of the cohort initiated ART at very low CD4 counts (50–200 cells). Whilst ART was recommended in all patients eligible for ART according to 2006 WHO guidelines [31] such patients with very low CD4 counts were prioritized and fast tracked for ART over those with higher CD4 counts as these usually have higher risk of dying. Slightly over half of the cohort did not have documentation of a baseline CD4 count test. Further analysis shows that these patients without CD4 counts did not constitute any specific sub-group of patients. Scale up of point-of-care CD4 count machines could greatly improve linkage to ART care and patient retention as they have been shown locally to be more suitable for immediate decision making, improve testing access in both rural and urban settings and can also be used by nurses when compared to conventional laboratory CD4 machines [32].

For those ART patients in Zimbabwe with documented follow-up data, there were some signs of immunologic and clinical improvement. Median gains in CD4+ cell counts at 6, 12, 24 and 36 months were similar to those reported from Rwanda [33], Mozambique [34] and Cote d'Ivoire [35] and other low- and high-income settings [36]. These immunologic gains in the absence of virological monitoring maybe highly associated with viral load suppression although it has recently been shown that immunologic monitoring coupled with monitoring viral load and duration on ART is very useful in assessing risk of patient mortality [37]. In our study the treatment failure rate which was based on a clinical/immunologic definition was lower than the average treatment failure rate of 2.64% reported for Africa in a systematic review by Renaud-Thiery F et al [38] when similarly based on the clinical/immunologic criteria. However, the clinical/immunologic criteria for treatment failure is known to be less sensitive in comparison to virological monitoring [39] and treatment failure rates were found to be approximately three times higher when viral load was used to define failure in the systematic review by Renaud-Thiery et al [38]. In Zimbabwe, findings from routine cohort monitoring at 12 sentinel sites for HIV drug resistance conducted between 2009 and 2011 show that 10.4% of 958 HIV-infected patients had viral load >1000 copies/ml at 12 months after starting ART, and upon excluding those with baseline gene mutations the proportion that had acquired HIV drug resistance was 8.9% [40]. This highlights a need for integration of viral load monitoring into routine ART care.

Table 4. Median CD4 T-cell count (cells/μL) gains during ART stratified by gender.

	6-month gains		12-month gains		24-month gains	
	**Median (IQR)**	***N***	**Median (IQR)**	***N***	**Median (IQR)**	***N***
All	122 (65–189)	222	157 (85–240)	279	252 (162–353)	162
Female	122 (68–193)	139	171 (89–252)	183	265 (180–367)	106
Male	125 (63–172)	83	130 (80–211)	96	179 (113–298)	56

Note: These analyses are not based on imputed data.
doi:10.1371/journal.pone.0086305.t004

Median gains in weight were also noted in this study and such observations during antiretroviral therapy have been associated with better patient outcomes [41]. Similar to our setting, the same has been reported in Rwanda [33] and Mozambique [34]. Routine recording of weight was notably a poor practice both at ART initiation and in follow up visits in this study and needs to be scaled up since WHO recommends monitoring weight [42] as a useful indicator of a patient's response to ART.

As found in other sub-Saharan African countries, retention of patients initiating ART at primary healthcare facilities in Zimbabwe was better than for those initiating ART at higher levels of care, in particular district/mission hospitals [10,11,43,44]. In further analysis we noted that there were more LFTUs in district/mission hospitals compared to primary care facilities. Whilst these LFTUs could have been silent deaths they could as well be patients who unofficially transferred from centralised services to PHCs where they can access better quality medical attention as found in a systematic review of mostly Sub-Saharan African studies [45]. During the study period under review, ART initiations were mostly led by doctors who were mostly situated in higher level health facilities due to their shortage. It is therefore likely that patients would travel from rural settings to district or mission hospitals, for ART initiations and there after unofficially transfer to other less congested peripheral facilities for follow-up care. This in part may overestimate attrition levels in these higher level facilities. To limit such potential biases, transfers-in were not sampled during data collection whilst official transfers-out where eliminated from the analysis.

This further suggests the importance of continuing the decentralization of OI/ART services through the accreditation of peripheral health facilities and outreach sites so that they can conduct ART initiations as described in the 2011 Zimbabwe Guidelines for the Decentralization of OI/ART Services [46]. This should also be coupled with endorsing nurse-led ART initiation, given the high shortage of medical doctors. In our view decentralisation improved patient retention in the National ART Programme, particularly at primary care facilities by decongesting ART initiations at higher level facilities although this may inevitably have contributed to high losses-to-care of patients from centralised facilities with high patient volumes who unofficially transferred to primary health facilities were they could access better quality of medical care and follow-up. To address future attrition we propose adoption of patient adherence groups for

Table 5. Factors associated with attrition including patient characteristics at ART initiation, clinical practices and healthcare facility type and location.

	Attrition						
	Original		Multiple Imputation (N = 3,919)				
	Frequency	*Rate/ 100PY*	*Rate/ 100PY*	*HR (95% CI)*	*p*-value	*AHR* (95% CI)	*p*-value
Sex							
Female	2,514	19.3	19.3	1.0		1.0	–
Male	1,393	24.0	24.0	**1.22 (1.07–1.39)**	**0.003**	**1.24 (1.08–1.43)**	**0.004**
CD4 T-cell count (cell/μL)							
>200	402	14.8	19.4	1.0	–	1.0	–
>50–≤200	1,031	13.2	19.5	1.03 (0.76–1.39)	0.861	0.97 (0.74–1.26)	0.806
<50	401	22.7	25.8	**1.36 (1.01–1.85)**	**0.045**	1.22 (0.89–1.67)	0.189
WHO Stage							
Stage I/II	390	15.8	17.5	1.0	–	1.0	–
Stage III	2,413	19.1	19.9	1.16 (0.76–1.77)	0.481	1.15 (0.74–1.78)	0.531
Stage IV	486	26.6	30.3	**1.73 (1.19–2.50)**	**0.006**	**1.73 (1.14–2.61)**	**0.012**
Weight							
>60 kgs	779	15.2	16.3	1.0	–	1.0	–
45–60 kgs	1,722	19.0	20.4	**1.25 (1.03–1.51)**	**0.024**	**1.25 (1.04–1.51)**	**0.020**
<45 kgs	369	33.4	34.7	**2.07 (1.53–2.79)**	**0.000**	**2.03 (1.43–2.87)**	**0.000**
Prescribed CTX							
Yes	2,985	16.7	20.3	1.0	–	1.0	–
No	109	34.5	39.5	**1.82 (1.22–2.72)**	**0.005**	**1.94 (1.21–3.09)**	**0.007**
Level of Healthcare							
Primary healthcare facility	415	8.4	8.4	1.0	–	1.0	–
District/Mission hospital	2,844	20.1	20.1	2.24 (0.81–6.20)	0.116	**3.49 (1.09–11.20)**	**0.036**
Central/Provincial hospital	660	29.8	29.8	3.16 (1.15–8.72)	**0.027**	**3.21 (0.96–10.75)**	**0.058**
Site Type							
Rural	1,975	14.8	14.8	1.0	–	1.0	–
Urban	1,944	27.8	27.8	**1.75 (0.99–3.09)**	**0.054**	**2.13 (0.95–4.77)**	**0.064**

doi:10.1371/journal.pone.0086305.t005

stable ART patients. In an urban setting in South Africa, these adherence groups have resulted in a 57% reduction in loss-to-care compared to those attending routine nurse-led care [47] whilst in rural Mozambique they have resulted in a remarkable 1-year patient retention of 97% [44].

As reported by other ART Programmes in sub-Saharan Africa, male sex and advanced disease at ART initiation were associated with patient death or loss to follow-up [33–35,48–50]. In our study, proportionally more women (64%) than men (36%) were on ART as reported by other Southern African countries [51]. This corresponds with national HIV testing patterns in Zimbabwe in which fewer women (42%) than men (62%) report never having been tested for HIV [13] and also with the observation that male HIV patients in sub-Saharan Africa are more likely than females to have advanced HIV disease at ART initiation and to be at increased risk of LTFU and/or death [49,52,53].

In many African settings, women have better access than men to HIV testing and treatment services through integration of routine "opt-out" provider-initiated testing and counselling in antenatal care settings [54] as an entry point into prevention-of-mother-to-child HIV transmission (PMTCT) services, even though attrition rates between testing HIV-positive in pregnancy and accessing ART continue to be unacceptably high [55,56]. A recent study by Takarinda and colleagues [57] suggests that even when men do have access to HIV testing and counselling they decline it. HIV testing rates can be scaled up through innovative strategies such as roll-out of routine opt-out mobile, home-based or work-place voluntary testing and counselling [58–60] which may improve male participation in order to achieve earlier HIV diagnosis and also provider initiated testing and counselling in a healthcare setting can improve testing for both genders. Strategies aimed at improving linkage to care from HIV testing may also help achieve earlier treatment and resultantly improve patient treatment outcomes.

A large percentage of the cohort had low body weight at baseline and consistent with other ART Programmes [33,34,49] the probability of retention was lower in patients with low body weight at ART initiation. Although it is not known whether undernourishment is causally related to poor ART outcomes or is merely associated [61], hunger is a frequently reported barrier to adherence and the side effects and toxicity of some ARVs can be potentiated if taken without food [62]. Adoption of food supplementation as a strategy aimed at increasing patient retention would be useful in our setting. This strategy has been shown to

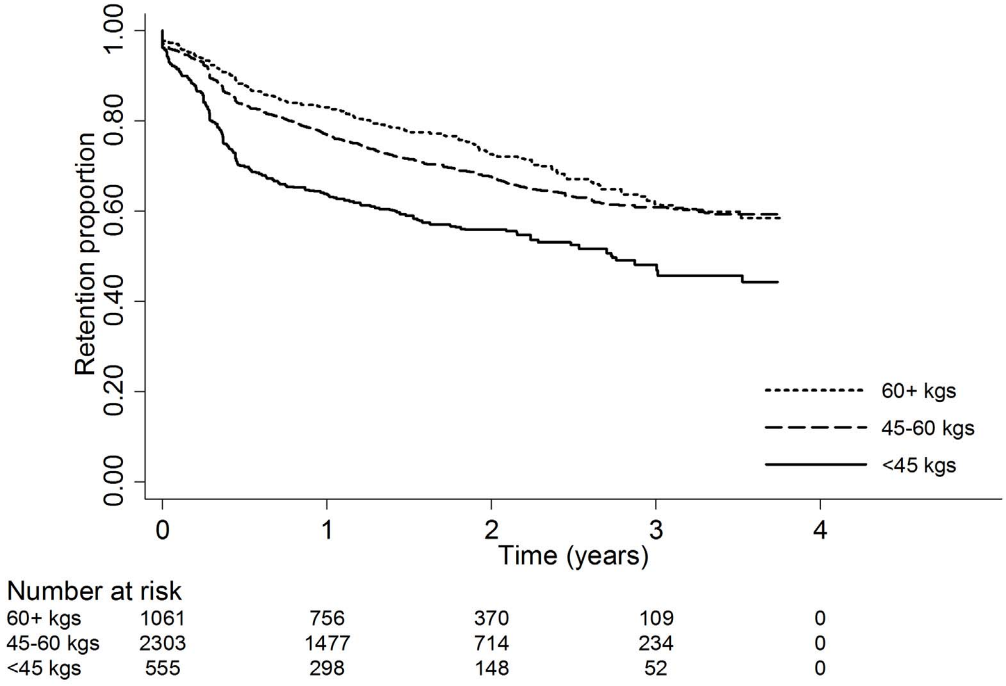

Figure 1. Retention stratified by weight at baseline.
doi:10.1371/journal.pone.0086305.g001

improve adherence to ART among food-insecure adults in Zambia [63] and also is associated with reduced morbidity and hospitalisations [64].

Anaemia has been shown to be an independent indicator of mortality and of disease progression among HIV-infected persons in sub-Saharan Africa [48,49,65,66]. Unfortunately, levels of anaemia could not be determined in our study because only 20.2% of patients had a documented haemoglobin level at baseline and even fewer had documented haemoglobin levels at follow-up visits. Zimbabwe has begun gearing up routine clinical laboratory testing for HIV-infected patients which should improve the capacity of clinicians to screen for anaemia.

Attrition was closely associated with advanced disease status at ART initiation as reported by ART programmes throughout sub-

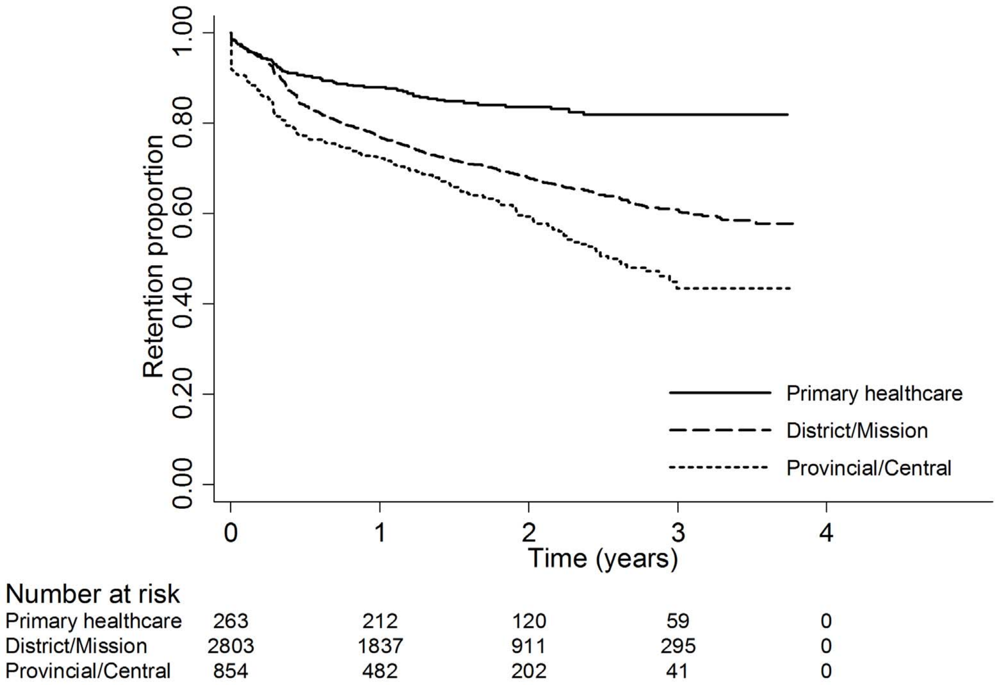

Figure 2. Retention stratified by level of healthcare.
doi:10.1371/journal.pone.0086305.g002

Saharan Africa [45,67]. Clinical predictors of attrition in Zimbabwe at initiation of ART included WHO clinical stage III and low body weight (<45 kg). Whilst those in WHO stage IV were at increased risk of attrition compared to those in WHO stage I/II, they constituted a small proportion of the cohort. The majority where in WHO stage III at ART initiation and although they were not significantly at higher risk of attrition, these patients had advanced HIV disease and where therefore also at increased risk of having poor outcomes compared to those in stage I/II. Data from South Africa and other countries demonstrate that starting ART earlier (at CD4<350 cells/μL) results in improved treatment outcomes [68]. Similar to other programmes [34,45,48,49], a large proportion of reported mortality in our study population occurred within 6 months of initiating ART. During the period of our study, adults infected with HIV were eligible for ART if they presented with WHO clinical stage IV and/or CD4+ <200 cells/μL. It is well known from literature that initiation of ART with poor health at baseline (specifically CD4 counts<200cells/ml or WHO stage IV or V) can lead to immune reconstitution inflammatory syndrome which can occur within days to months from ART initiation and has clinical effects ranging from a mild, self-limiting illness to severe morbidity and mortality. [69,70] Our findings underscore the importance of initiating treatment at an earlier disease stage as recommended by the recently published 2013 WHO Consolidated HIV Guidelines [71].

The 2013 Consolidated HIV Guidelines present opportunities for Zimbabwe to provide early HIV treatment and care services for HIV infected people using a higher CD4 threshold of <500. In 2013 alone, the number of people in need of treatment will increase by 25% from 685,360 (based on the 2010 guidelines) to 857,842 [12]. It is likely that the country will adopt Tenofovir +Lamivudine+Efavirenz, as the preferred 1st line ART regimen for adults and adolescents. Although more costly than stavudine-based regimens; it is hoped that as more countries adopt the guidelines and order large volumes of medicines, the drug price for tenofovir-based regimens will decline over time with economies of scale. It is feasible though that Zimbabwe will successfully adopt and implement the 2013 WHO HIV guidelines using the recently approved Global Fund HIV grant of USD 555 million.

Despite our study having a large sample which enables us to confidently infer our findings to the entire national ART programme, there were limitations to our study. These limitations were mainly related to data abstraction of routinely collected programme data hence this accounted for missing data in a number of variables collected. We were also unable to establish true patient outcomes for those patients that were classified as LFTU or defaulters. Also the exclusion of patients that transferred out or transferred into the selected ART sites may limit inference of our findings to this patient group although further comparison of the abstracted "transfer-out" patient group to the rest of the cohort showed no differences in baseline characteristics. The above mentioned limitations may therefore lead to biased estimates on patient retention and attrition-associated factors in the national patient cohort.

Conclusion

The assessment covered a difficult period for Zimbabwe and despite this, the rapid scale-up of ART has been remarkable. Also whilst retention and clinical outcomes are comparable or better than those reported from programmes in other sub-Saharan African countries, there is still need to monitor and improve on retention, and some strategies may include the adoption of ART adherence clubs [43] and issuing out of mobile phone short message services as drug pick-up and clinic visit reminders to patients [72] as shown in other settings. Adoption of food supplementation can be another feasible strategy to improve patient retention among food insecure adults.

In addition, our findings indicate that increased efforts are needed to ensure that persons with HIV seek care and initiate treatment before they have advanced disease and to expand access of services through decentralisation and targeted services (e.g., for men).

HIV testing rates and linkages to care, especially for men, need to be improved (perhaps through the provision of services in non-traditional venues) in order to achieve earlier diagnosis and treatment whilst CD4 cell count testing needs to be scaled up at district level to assess ART eligibility [55,66] and this can be through the use of CD4 point-of-care machines.

Acknowledgments

The authors would like to acknowledge those who implemented data abstraction, data cleaning, and data entry activities: Gibson Chikono, Millicent Chilongo, Luke Chimhanda, Priscilla Chirisa, Yvonne Chitate, Somkie Dube, Janet Dzangare, Harusekwi Andrew Hukuimwe, Wendy Inouye, Nhamo Jaravaza, Lydia Madyira, Alpha Mukaronda, Cansio Makombe, D Mambudzi, Judith Mbirimi, Richard Mashapa, Alice Masoha, Absolom Mbinda, Winter Mkwananzi, M Moyo, Thomas Moyo, Christopher Muchira, Maclean Mukombachoto, Pardon Munharira, Rutendo Munharira, Mirirai Muyengwa, John Nyamayaro, Tendai Zimunya.

Disclaimer

The findings and conclusions in this manuscript are those of the authors and do not necessarily represent the views of the US Centers for Disease Control and Zimbabwe Government.

Author Contributions

Conceived and designed the experiments: TMA RWS AA. Performed the experiments: TMA RWS JD AA CJW. Analyzed the data: RWS KCT JD. Contributed reagents/materials/analysis tools: TMA RWS AA KCT JD. Wrote the paper: TMA RWS KCT JD OM JM AA CJW.

References

1. WHO, UNICEF, UNAIDS (2013) Global update on HIV treatment 2013: results, impact and opportunities, June 2013 Brief summary. Kuala Lumpur: WHO, UNICEF, UNAIDS. p. 7–19. Available: http://apps.who.int/iris/bitstream/10665/85327/1/WHO_HIV_2013.9_eng.pdf. Accessed 2013 Oct 12.
2. United Nations General Assembly (2011) 2011 Political Declaration on HIV and AIDS: Intensifying Our Efforts to Eliminate HIV and AIDS. New York: United Nations General Assembly. p. 8. Available: http://www.unaids.org/en/media/unaids/contentassets/documents/document/2011/06/20110610_un_a-res-65-277_en.pdf. Accessed 2013 Oct 12.
3. Geng EH, Nash D, Kambugu A, Zhang Y, Brainstein P, et al. (2010) Retention in care among HIV-infected patients in resource-limited settings: emerging insights and new directions. Curr HIV/AIDS Rep 7: 234–244.
4. Fox MP, Rosen S (2010) Patient retention in antiretroviral therapy programs up to three years on treatment in sub-Saharan Africa, 2007–2009: systematic review. Trop Med Int Health 15: 1–15.
5. Lambdin BH, Micek MA, Koepsell TD, Hughes JP, Sherr K, et al. (2011) Patient volume, human resource levels, and attrition from HIV treatment programs in central Mozambique. J Acquir Immune Defic Syndr 57: e33–39.
6. Bussmann H, Wester CW, Ndwapi N, Grundmann N, Gaolathe T, et al. (2008) Five-year outcomes of initial patients treated in Botswana's National Antiretroviral Treatment Program. AIDS 22: 2303–2311.
7. WHO, UNAIDS, UNICEF (2011) Global HIV/AIDS response: epidemic update and health sector progress towards universal access: progress report 2011. Geneva: WHO. p. 93–95. Available: http://whqlibdoc.who.int/publications/2011/9789241502986_eng.pdf. Accessed 2013 Oct 12.

8. Bemelmans M, Akker TVD, Ford N, Phillips M, Zachariah R, et al. (2010) Providing universal access to antiretroviral therapy in Thyolo, Malawi through task shifting and decentralization of HIV/AIDS care. Trop Med Int Health 15:1413–1420.
9. Boyer S, Eboko F, Camara M, Abé C, Nguini ME, et al. (2010) Scaling up access to antiretroviral therapy for HIV infection: the impact of decentralization of healthcare delivery in Cameroon. AIDS 24: S5–S15.
10. Chan AK, Matevu G, Jahn A, Schouten E, Arora P, et al. (2010) Outcome assessment of decentralization of antiretroviral therapy provision in a rural district of Malawi using an integrated primary care model. Trop Med Int Health 15: 90–97.
11. Fatti G, Grimwood A, Bock P (2010) Better antiretroviral therapy outcomes at primary healthcare facilities: an evaluation of three tiers of ART services in four South African provinces. PLoS One 5: e12888.
12. Ministry of Health and Child Welfare (MOHCW) (2013) Zimbabwe HIV Estimates, 2012. Harare: MOHCW. p. 17–19.
13. Zimbabwe National Statistics Agency (ZIMSTAT) and ICF International (2012) Zimbabwe Demographic and Health Survey 2010–2011. Calverton, Maryland: ZIMSTAT and ICF International. p. 220.
14. Meldrum A (2008) Zimbabwe's health care system struggles on. Lancet 371: 1059–1060.
15. Ministry of Health and Child Welfare (MOHCW) (2006) Zimbabwe ART annual report 2006. Harare: MOHCW. p. 49.
16. Joint United Nations Programme on HIV/AIDS (UNAIDS). (2008) Report on the global HIV/AIDS epidemic 2008. Geneva: UNAIDS. p. 272. Available: http://www.unaids.org/en/media/unaids/contentassets/dataimport/pub/globalreport/2008/jc1510_2008globalreport_en.pdf. Accessed 2013 Oct 31.
17. Ministry of Health and Child Welfare (MOHCW) (2009) Zimbabwe ART annual report 2009. Harare: MOHCW. p.15
18. Joint United Nations Programme on HIV/AIDS (UNAIDS) (2010) Global report: UNAIDS report on the global AIDS epidemic 2010. Geneva: UNAIDS. p. 253. Available: http://www.unaids.org/documents/20101123_globalreport_em.pdf. Accessed 2013 Oct 31.
19. Mills EJ, Nachega JB, Buchan I, Orbinski J, Attaran A, et al. (2006) Adherence to antiretroviral therapy in sub-Saharan Africa and North America: a meta-analysis. JAMA 296: 679–690.
20. Nachega JB, Mills EJ, Schechter M (2010) Antiretroviral therapy adherence and retention in care in middle-income and low-income countries: current status of knowledge and research priorities. Curr Opin HIV AIDS 5: 70–77.
21. Fehd RJ (1998) %ComparWS: Compare with summary: a macro using Proc Compare to write a file of differences to edit and use for updates. Available: http://www2.sas.com/proceedings/sugi23/Posters/p170.pdf. Accessed 2013 Oct 31.
22. Ministry of Health and Child Welfare (MOHCW) (2008) Zimbabwe ART annual report 2008. Harare: MOHCW. p. 17.
23. Rubin DB (1987) Multiple imputation for nonresponse in surveys. New York: Wiley. 258 p.
24. Royston P (2004) Multiple imputation of missing values. The Stata Journal 4: 227–241.
25. Royston P (2005) Multiple imputation of missing values: update. The Stata Journal 5: 188–201.
26. Royston P (2005) Multiple imputation of missing values: update of ice. The Stata Journal 5: 527–536.
27. Royston P, Carlin JB, White IR (2009) Multiple imputation of missing values: new features for mim. The Stata Journal 9: 252–264.
28. White IR, Royston P (2009) Imputing missing covariate values for the Cox model. Statist Med 28: 1982–1998.
29. Hanke SH (2012) Zimbabwe: From hyperinflation to growth. Available: http://object.cato.org/sites/cato.org/files/pubs/pdf/dpa6.pdf. Accessed 2013 Oct 15.
30. Ministry of Health and Child Welfare and WHO Zimbabwe Country office (2012) The Expanded Support Programme for HIV and AIDS (ESP) in Zimbabwe, Treatment and Care Component: May 2007–March 2012 End of Project Report. Harare: MOHCW.
31. Gilks C, Vitoria M, World Health Organisation (WHO). Dept. of HIV/AIDS (2006) Antiretroviral therapy for HIV infection in adults and adolescents: recommendations for a public health approach. 2006 revision. Geneva: WHO. p. 13–16. Available: https://extranet.who.int/iris/restricted/bitstream/10665/43554/1/9789241594677_eng.pdf. Accessed 2013 Oct 31.
32. Mtapuri-Zinyowera S, Chideme M, Mangwanya D, Mugurungi O, Gudukeya S, et al. (2010) Evaluation of the PIMA point-of-care CD4 analyzer in VCT clinics in Zimbabwe.J Acquir Immune Defic Syndr; 55: 1–7.
33. Lowrance DW, Ndamage F, Kayirangwa E, Ndagije F, Low W, et al. (2009) Adult clinical and immunological outcomes of the national antiretroviral treatment program in Rwanda during 2004–2005. J Acquir Immune Defic Syndr 52: 49–55.
34. Auld AF, Mbofana F, Shiraishi RW, Sanchez M, Alfredo C, et al. (2011) Four-year treatment outcomes of adult patients enrolled in Mozambique's rapidly expanding antiretroviral therapy program. PLoS ONE 6: e18453.
35. Toure S, Kouadio B, Seyler C, Traore M, Dakoury-Dogbo N, et al. (2008) Rapid scaling-up of antiretroviral therapy in 10,000 adults in Cote d'Ivoire: 2-year outcomes and determinants. AIDS 22: 873–882.
36. Braitstein P, Brinkhof MW, Dabis F, Schechter M, Boulle A, et al. (2006) Mortality of HIV-1-infected patients in the first year of antiretroviral therapy: comparison between low-income and high-income countries. Lancet 367: 817–824.
37. Brennan AT, Maskew M, Sanne I, Fox MP, et al. (2013) The interplay between CD4 cell count, viral load suppression and duration of antiretroviral therapy on mortality in a resource-limited setting. Trop Med Int Health 18: 619–631.
38. Renaud-Thery F, Duncombe C, Kerr S, Thierry S, Perriëns J, et al. (2010) Adult antiretroviral therapy in resource limited settings: a systematic review of first-line failure and attrition rates. Available: http://www.who.int/hiv/topics/treatment/First_Line_ART_failure_RLS_metanalysis.pdf. Accessed 2013 Oct 28.
39. Moore DM, Mermin J, Awor A, Yip B, Hogg RS, et al. (2006) Performance of immunologic responses in predicting viral load suppression: implications for monitoring patients in resource-limited settings. J Acquir Immune Defic Syndr 43: 436–439.
40. Ministry of Health and Child Welfare (MOHCW) (2012) Report on the National HIV Drug Resistance Monitoring at Sentinel sites (2009–2011). Harare: MOHCW. p. 41.
41. Madec Y, Szumilin E, Genevier C, Ferradini L, Balkan S, et al. (2009) Weight gain at 3 months of antiretroviral therapy is strongly associated with survival: Evidence from two developing countries. AIDS 23: 853–861.
42. World Health Organization (WHO) Dept. of HIV/AIDS, WHO Integrated Management of Adolescent and Adult Illness Project, WHO Integrated Management of Childhood Project (2007) Chronic HIV care with ART and prevention: Integrated Management of Adolescent and Adult Illness, Integrated Management of Childhood Illness interim guidelines for health workers at health centre or district hospital outpatient clinic. Geneva: WHO. p. 61. Available: https://extranet.who.int/iris/restricted/bitstream/10665/43680/1/9789241595513_eng.pdf Accessed 2013 Oct 25.
43. Bedelu M, Ford N, Hilderbrand K, Reuter H (2007) Implementing antiretroviral therapy in rural communities: the Lusikisiki model of decentralized HIV/AIDS care. J Infect Dis 196: 464–468.
44. Decroo T, Panunzi I, das Dores C, Maldonado F, Biot M, et al. (2009) Lessons learned during down referral of antiretroviral treatment in Tete, Mozambique. J Int AIDS Soc 12: 6.
45. Brinkhof MWG, Pujades-Rodriguez M, Egger M (2009) Mortality of Patients Lost to Follow-Up in Antiretroviral Treatment Programmes in Resource-Limited Settings: Systematic Review and Meta-Analysis. PLoS ONE 4: e5790.
46. Ministry of Health and Child Welfare (MOHCW) (2011) Zimbabwe Guidelines for the Decentralization of OI/ART Services. Harare: MOHCW. p. 12.
47. Luque-Fernandez MA, Van Cutsem G, Goemaere E, Hilderbrand K, Schomaker M, et al. (2013) Effectiveness of Patient Adherence Groups as a Model of Care for Stable Patients on Antiretroviral Therapy in Khayelitsha, Cape Town, South Africa. PLoS ONE 8: e56088.
48. Stringer JS, Zulu I, Levy J, Stringer EM, Mwango A, et al. (2006) Rapid scale-up of antiretroviral therapy at primary care sites in Zambia: feasibility and early outcomes. JAMA 296: 782–793.
49. May M, Boulee A, Phiri S, Messou E, Myer L, et al. (2010) Prognosis of patients with HIV-1 infection starting antiretroviral therapy in sub-Saharan Africa: a collaborative analysis of scale-up programmes. Lancet 376: 449–457.
50. Ekouevi DK, Balestre E, Ba-Gomis FO, Eholie SP, Maiga M, et al. (2010) Low retention of HIV-infected patients on antiretroviral therapy in 11 clinical centres in West Africa. Trop Med Int Health 15:34–42.
51. Muula AS, Thabale JN, Siziya S, Makupe CM, Umar E, et al. (2007) Gender distribution of adult patients on highly active antiretroviral therapy in southern Africa. BMC Public Health 7: 63.
52. Mills EJ, Bakanda C, Birungi J, Chan K, Hogg RS, et al. (2011) Male gender predicts mortality in a large cohort of patients receiving antiretroviral therapy in Uganda. J Int AIDS Soc 14: 52.
53. Hawkins C, Chalamilla G, Okuma J, Spiegelman D, Hertzmark E, et al. (2011) Sex differences in antiretroviral treatment outcomes among HIV-infected adults in an urban Tanzanian setting. AIDS 25: 1189–1197.
54. Staveteig S, Wang S, Head SK, Bradley SEK, Nybro E (2013) Demographic Patterns of HIV Testing Uptake in Sub-Saharan Africa. DHS Comparative Reports No. 30. Calverton, Maryland, USA: ICF International. p. 39–47. Available: http://www.measuredhs.com/pubs/pdf/CR30/CR30.pdf. Accessed 2013 Oct 28.
55. Ferguson L, Grant AD, Watson-Jones D, Kahawita T, Ong'ech JO, et al. (2012) Linking women who test HIV-positive in pregnancy-related services to long-term HIV care and treatment services: a systematic review. Trop Med Int Health 17: 564–580.
56. Muchedzi A, Chadambuka A, Chikwinya B, Mahomva A (2012) Evaluating the effect of the use of point-of-care CD4 machines on access to antiretroviral therapy (ART) eligibility screening and ART initiation for HIV-positive pregnant women in Zimbabwe: towards elimination of new paediatric HIV infection by 2015.: 19th International AIDS Conference: Abstract no. -TUPDE0204. Available: http://iasociety.org/Abstracts/A200745824.aspx. Accessed 2013 Oct 31.
57. Takarinda K, Harries AD, Srinath S, Mutasa-Apollo T, Sandy C, et al. (2012) Treatment outcomes of adult patients with recurrent tuberculosis in relation to HIV status in Zimbabwe: A retrospective record review. BMC Public Health 12: 124.
58. Matovu JKB, Makumbi FE (2007) Expanding access to voluntary HIV counselling and testing in sub-Saharan Africa: alternative approaches for improving uptake, 2001–2007. Trop Med Int Health 12: 1315–1322.

59. Corbett EL, Dauya E, Matambo R, Cheung YB, Makamure B, et al. (2006) Uptake of Workplace HIV Counselling and Testing: A Cluster-Randomised Trial in Zimbabwe. PLoS Med 3: e238.
60. Kuwane B, Appiah K, Felix M, Grant A, Churchyard G (2009) Expanding HIV care in Africa: making men matter in Johannesburg. Lancet 374: 1329.
61. Koethe JR, Heimburger DC (2010) Nutritional aspects of HIV-associated wasting in sub-Saharan Africa. Am J Clin Nutr 91: 1138S–1142S.
62. Koethe JR, Chi BH, Megazzini KM, Heimburger DC, Stringer JS, et al. (2009) Macronutrient supplementation for malnourished HIV-infected adults: A review of the evidence in resource-adequate and resource-constrained settings. Clin Infect Dis 49: 787–798.
63. Cantrell RA, Sinkala M, Megazinni K, Lawson-Mariott S, Washington S, et al. (2008) A pilot study of food supplementation to improve adherence to antiretroviral therapy among food-insecure adults in Lusaka, Zambia. J Acquir Immune Defic Syndr 49: 190–195.
64. Weiser SD, Hatcher A, Frongillo EA, Guzman D, Riley ED, et al. (2013) Food insecurity is associated with greater acute care utilization among HIV-infected homeless and marginally housed individuals in San Francisco. J Gen Intern Med 28: 91–98.
65. O'Brien ME, Kupka R, Msamanga GI, Saathoff E, Hunter DJ, et al. (2005) Anemia is an independent indicator of mortality and immunologic progression of disease among women with HIV in Tanzania. J Acquir Immun Defic Syndr 40: 219–225.
66. Ojikutu BO, Zheng H, Walensky RP, Lu Z, Losina E, et al. (2008) Predictors of mortality in patients initiating antiretroviral therapy in Durban, South Africa. S Afr Med J 98: 204–208.
67. Lawn SD, Harries AD, Wood R (2010) Strategies to reduce early morbidity and mortality in adults receiving antiretroviral therapy in resource-limited settings. Curr Opin HIV AIDS 5: 18–26.
68. Fox MP, Sanne IM, Conradie F, Zeinecker J, Orrell C, et al. (2010) Initiating patients on ART at CD4 counts above 200 is associated with improved treatment outcomes in South Africa. AIDS 24:2041–2050.
69. Hirsh HH, Kaufmann G, Sendi P, Battegay M (2004) Immune Reconstitution in HIV-Infected Patients. Clin Infect Dis 38: 1159–1166.
70. Elston WT, Thaker H (2009) Immune reconstitution inflammatory syndrome. Int J STD AIDS 2009; 20: 221–224.
71. World Health Organisation (WHO) (2013) Consolidated guidelines on the use of antiretroviral drugs for treating and preventing HIV infection: recommendations for a public health approach. Geneva: WHO. Available: http://apps.who.int/iris/bitstream/10665/85321/1/9789241505727_eng.pdf Accessed 2013 Oct 27.
72. Lester RT, Ritvo P, Mills EJ, Kariri A, Karanja S, et al. (2010) Effects of a mobile phone short message service on antiretroviral treatment adherence in Kenya (WelTel Kenya1): a randomised trial. Lancet 376: 1838–1845.
