# Supplementary material for: Retention and predictors of attrition among patients who started antiretroviral therapy in Zimbabwe’s national antiretroviral therapy programme between 2012 and 2015
Source: PLoS One. 2020 Jan 7;15(1):e0222309. doi: 10.1371/journal.pone.0222309 (PMC6946589; doi:10.1371/journal.pone.0222309)
Supplement: S1 Table — (DOCX) [file pone.0222309.s002.docx]

**S1 Table: Follow-up time of the 3810 patients who started ART in Zimbabwe between 2012 – 2015**

| **Year** | **Observations** | **Observed follow-up time in months** | |
| --- | --- | --- | --- |
|  |  | **Minimum** | **Maximum** |
| 2012 | 289 | 0.09 | 40.4 |
| 2013 | 1476 | 0.1 | 37.2 |
| 2014 | 1850 | 0.1 | 25.2 |
| 2015 | 195 | 0.1 | 13.4 |
