# Supplementary material for: Retention and predictors of attrition among patients who started antiretroviral therapy in Zimbabwe’s national antiretroviral therapy programme between 2012 and 2015
Source: PLoS One. 2020 Jan 7;15(1):e0222309. doi: 10.1371/journal.pone.0222309 (PMC6946589; doi:10.1371/journal.pone.0222309)
Supplement: Attachment — Submitted filename: Letter PLOS_submitted20191017.doc [file pone.0222309.s003.doc]

**Reviewer Recommendation and Comments for Manuscript Number PONE-D-19-23547**

This is a large retrospective study evaluating the risk of LTFU among individuals who started ART between 2012-2015 in Zimbabwe. We commend the efforts of working with program data to produce this manuscript and support it’s publication.

I believe this study will benefit from the following comments:-

1. The authors should be more specific about the purpose of this study, the comparisons and the hypotheses.
2. How would patients who were considered LTFU (>180 days since without a visit) but show up at the clinic over 6 months later were treated in the analysis? I.e. are there some cases that were LFTU based on this criteria but showed up after 6 months has lapsed?
3. Could the other program activities (Line 294) explain lower retention in the 2014-2015 cohort compared to 2012-2013? (Fig 3). Did these activities diminish over time? I recall reading that more individuals initiated ART over time so does it mean that although ART was scaled up, the retention was lower. Please correct my misinterpretations and kindly explain these results.
4. Discuss program adherence and retention interventions (if any) and consider citing a paper by Coker et al., Curr HIV/AIDS Reports 2015. Socio-Demographic and Adherence Factors Associated with Viral Load Suppression in HIV-Infected Adults Initiating Therapy in Northern Nigeria: A Randomized Controlled Trial of a Peer Support Intervention that highlights that indicate that ART adherence will improve significantly regardless of whether HIV-infected adults received adherence interventions or standard of care services.
5. How did the authors approach to missing data? Working with this large amount of program data will no doubt be challenged with missingness with respect to clinical parameters such as CD4, WHO etc. The authors discussed that CD4 testing was became available in 63 districts in 2015 compared to 47 in 2009 (Line 146-147). The authors may consider using modern approaches to missing data, including multiple imputation or evaluating how the types of missingness.
6. Did the authors assess pharmacy refill adherence?
7. There was discussion about higher attrition in PHCs. Were there any significant differences in retention across districts/clinics/sites?
8. The lower risk of attrition in children and adolescents is good news and merits some discussion.
9. Consider comparing patients who were LTFU (not retained) with those that were retained in care based on variables such as baseline CD4, etc. Consider including in Table 3
10. Figure 1 and 2: need higher resolution
