# Supplementary material for: Retention and predictors of attrition among patients who started antiretroviral therapy in Zimbabwe’s national antiretroviral therapy programme between 2012 and 2015
Source: PLoS One. 2020 Jan 7;15(1):e0222309. doi: 10.1371/journal.pone.0222309 (PMC6946589; doi:10.1371/journal.pone.0222309)
Supplement: Attachment — Submitted filename: Response to Reviewers.docx [file pone.0222309.s004.docx]

Dear Editor,

Many thanks for the review of “Retention and predictors of attrition among patients who started antiretroviral therapy in Zimbabwe’s National Antiretroviral Therapy Programme between 2012 and 2015". We have carefully read and discussed the recommendations made by the reviewers, and have adapted the paper accordingly.

A marked-up copy of the manuscript highlighting changes made to the original version labeled 'Revised Manuscript with Track Changes' and a clean version labeled 'Manuscript' have been uploaded.

In this response letter we respond to each point raised by the reviewers. Please find our responses below with revisions or additions to the manuscript in quotes. We shall of course be available to respond to any additional question you may have.

Kind regards,

Richard Makurumidze, on behalf of the co-authors

**Reviewer recommendation and comments for manuscript number PONE-D-19-23547**

**Reviewer 1**

This is a large retrospective study evaluating the risk of LTFU (Lost to follow-up) among individuals who started ART between 2012-2015 in Zimbabwe. We commend the efforts of working with program data to produce this manuscript and support its publication.

I believe this study will benefit from the following comments:-

1. **The authors should be more specific about the purpose of this study, the comparisons and the hypotheses.**

This retrospective follow-up study was not hypothesis driven and was independent from the previous evaluation though there were similarities in the methodology. Nevertheless, we had expected an increase of retention on ART due to the interventions recommendations from the previous evaluation. We mention this in the revised final paragraph of the introduction. The revised aim is shown in the last phrase of the introduction.

We revised and expanded the last part of the introduction as follows;

“We expected an increase in retention on ART following interventions recommended after the previous evaluation . The aim was to estimate retention, compare it with the previous evaluation, and explore predictors of attrition (either lost to follow-up or died).” (lines 109-112)

2. **How would patients who were considered LTFU (>180 days since without a visit) but show up at the clinic over 6 months later were treated in the analysis? I.e. are there some cases that were LTFU based on this criteria but showed up after 6 months has lapsed?**

We have no data on the number of patients that reengaged after being LTFU. We assessed the proportion LTFU, using the definition shown, on the date of data abstraction.

*We expanded the methods as follows:*

*“*The data was collected manually from the patient files.”( line 172)

“The patient status was determined on the date of data abstraction, regardless of previous treatment interruptions.” ( lines 178-180)

3. **Could the other program activities (Line 294) explain lower retention in the 2014-2015 cohort compared to 2012-2013? (Fig 3). Did these activities diminish over time? I recall reading that more individuals initiated ART over time so does it mean that although ART was scaled up, the retention was lower. Please correct my misinterpretations and kindly explain these results.**

Indeed, retention was lower among those who started ART between 2014-2015. This has been discussed citing change in ART initiation criteria, which included roll out of Option B+, as the main reason for the difference (lines 410-420). In addition, decentralization may have affected retention. Decentralization gradually increased between the period from 2012 to 2015. By the end of 2015 more patients were receiving care at PHCs, and our study found attrition to be higher at PHCs. True, the number of patients initiated on ART increased over time with a majority of them being initiated and followed-up at PHCs. In the discussion we speculate that the gradual increase over time of patients starting ART at the possibly ill-prepared PHCs might have also contributed to the increase in attrition during the study period. As discussed (lines 302-305) other adherence strategies were not implemented on a wide scale. A recommendation for further studies to understand this increase in attrition has been added (lines 447-448).

We have expanded the discussion as follows,

“Decentralization happened gradually between 2012- 2015. By the end of 2015 more patients were receiving care at PHCs, and our study found attrition to be higher at PHCs. The number of patients who were being initiated and followed-up on ART at PHCs increased during the period (Figure 1). We speculate that the gradual increase of patients starting ART at the possibly ill-prepared PHCs might have contributed to the increase in attrition over time during the study period.” (lines 405- 410)

4. **Discuss program adherence and retention interventions (if any) and consider citing a paper by Coker et al., Curr HIV/AIDS Reports 2015. Socio-Demographic and Adherence Factors Associated with Viral Load Suppression in HIV-Infected Adults Initiating Therapy in Northern Nigeria: A Randomized Controlled Trial of a Peer Support Intervention that highlights that indicate that ART adherence will improve significantly regardless of whether HIV-infected adults received adherence interventions or standard of care services.**

*,*

Widely implemented interventions that could have affected adherence and/or retention were decentralization and early ART initiation. Other interventions recommended by the prior evaluation, such as adherence clubs, food supplementation and mobile short messages service (SMS) reminders, were not implemented on a wider scale. Hence, these were not discussed in detail.

Thank you for the reference article by Coker et al (2015). From our understanding, the paper looked at home-based adherence and retention interventions i.e. use of lay people (peer educators and home-based treatment partners) to provide retention and adherence support in the HIV-infected person’s home. These activities were not widely implemented in Zimbabwe during the study period. The article also showed that males were less likely to be retained in care at the end of the follow-up period which is in contrast to our finding. We cited the article, using reference number 39.

5. **How did the authors approach to missing data? Working with this large amount of program data will no doubt be challenged with missingness with respect to clinical parameters such as CD4, WHO etc. The authors discussed that CD4 testing was became available in 63 districts in 2015 compared to 47 in 2009 (Line 146-147). The authors may consider using modern approaches to missing data, including multiple imputation or evaluating how the types of missingness.**

From the start we were aware of challenges related to missing data as we planned to work with such a large program database. Our sample size calculation was adjusted to cater for missing data, i.e. we assumed 20% missingness (3200 was required without adjusting for missingness). Patients without outcome data were excluded. Explanatory variables which had more than 30% missingness were excluded from the analysis. The data were assumed to be missing at random. Categorical variables which had less than 30% missingness were included in analysis (Table 1). We used the missing indicator method, showing the proportion missingness for the different variables (if any), and avoiding that observations were excluded from multivariable analysis.

The methods sections has been expanded as follows;

“Our sample size calculation was adjusted to cater for missing data. Patients without outcome data were excluded. Explanatory variables which had more than 30% missingness were also excluded from the analysis. The data were assumed to be missing at random. Categorical variables which had less than 30% missingness were included in the analysis (Table 1). We used the missing indicator method, showing the proportion missingness for the different variables (if any).”(lines 198-202)

The limitations section was expanded as follows;

“To preserve all observations for variables with less than 30% missingness the missing indicator method was used. We assumed that missingness was at random, and mainly explained by health workers inconsistently completing the medical records. However, the method can affect the residual variance of the regression” (lines 433- 436).

6. **Did the authors assess pharmacy refill adherence?**

Yes. We expanded the methods section as follows:

*“*If a patient was reported as LTFU in the patient file, data collectors checked pharmacy refill records to confirm LTFU. Refill dates were considered as visit dates.” (lines 181-183)

7. **There was discussion about higher attrition in PHCs. Were there any significant differences in retention across districts/clinics/sites?**

Receiving care at PHC level was a risk factor for attrition. Our paper focused on assessing risk factors and outcomes at patient level. We acknowledge that there might be differences in terms of retention at site/clinic/district level and recommend further analysis with level of care as a unit of analysis. We plan to use mixed methods to identify differences between clinics, and to describe experiences and practices of clinics performing better or worse than others (line 446).

In the limitation section we expanded as follows:

“Moreover, we did not assess differences between clinics of the same level of care.”(lines 436-437 )

8. **The lower risk of attrition in children and adolescents is good news and merits some discussion**.

Many thanks. We expanded the discussion as follows:

“The previous evaluation did not include data on children (< 15 years) but included adolescents (>= 15 years) and young adults. Though age was included the previous evaluation did not assess the effect of age on attrition [17]. In our study we found children (< 15 years) to be at lower risk of attrition as compared to adults. Our findings seem to contrast with those from previous studies, which identified dependency of guardians, lack of palatable formulations, and challenges with disclosure as additional challenges experienced by children [42–44]. We speculate that the following interventions impacted positively on retention in our setting: PMTCT Option B+ targeting the mother-baby pair, integration of paediatric ART services with immunisation, decentralization of paediatric ART services, improved access to palatable paediatric ART formulations, and emphasis on secondary caregivers to support the primary caregivers with the child’s adherence in case of absence or other commitments [45–48].” (lines 362-372)

9. **Consider comparing patients who were LTFU (not retained) with those that were retained in care based on variables such as baseline CD4, etc. Consider including in Table 3**

Many thanks for this suggestion for us to consider comparing baseline characteristics among those retained and LTFU. In our paper we have already compared these characteristics using the hazard ratios and attrition (those not retained) are shown proportions and person-years follow-up time. (Table 3). We have also structured Table 3 in a format similar previous evaluation for easy comparison. Baseline CD4 was not included as part of baseline characteristics because it was missing among 45.4% of study participants. We mention this in the limitations;

“We could not report on immunologic and virologic patient outcomes due to missing information in the patient manual medical records. Information on viral load testing and CD4 testing was more than 30% missing ” (lines 430-432)

10. **Figure 1 and 2: need higher resolution**

We have tried to adjust the resolution.

**Second Reviewer**

1. **Sample size: Your assumptions regarding the sample size does not include the comparison between the retention rates at 12 months between the actual and the former study. Reading the section it seems you need 4000 people to estimate a single rate – but then you should maybe give the broadness of a confidence interval to motivate this sample size. What do you mean by design effect 2.0?**

The sample size calculation method described was for the current evaluation without taking into consideration the comparison. This retrospective follow-up study was independent from the prior evaluation though there are similarities in the methodology. The comparison between the previous and the present study was a post hoc analysis.

Our primary goal was estimating the retention rate of the current period, hence the sample size was directly calculated for this, aiming for a maximum error of 5% (line 156) – which is directly linked to the width of the CI. While technically possible, calculating a sample size for the comparison would have been very imprecise and counterproductive as we did not have an a priori idea of the magnitude of the observed difference in retention that would be found. Given (1) the maximally 5% error on the retention estimate in the current period, (2) the known error of the retention in the previous period and (3) independence of results from both time periods, we were confident that we’d have the power to detect relevant substantial changes. The design effect of 2 was used to correct the loss of effective sample due to anticipated intra-stratum correlation. The sample size and sampling criteria section was expanded as follows,

“The design effect of 2 was used to correct the loss of effective sample which was anticipated from intra-stratum correlation.” (lines 156-158)

2. **Sampling criteria: You sampled from 1.10.2012 to 31.12.2015. The analysis was done in 2016? When I see this information I conclude that in the sub-cohort of those persons starting the ART-Program in 2014/15 there is nobody that can have a retention until 36 months (if starting 2014) and 24 months (if starting in 2015). I don’t find any information at the minimum/maximum length of observation in the (sub-) cohort, could you please add this.**

Yes, data was abstracted in February 2016. The difference in maximums was not a problem as there was no time-dependency (i.e. there was a constant hazard over time).We added the following table (Supplementary Table 1), to which we refer in the results section as follows:

“ The maximum follow-up time for the patients who started ART in 2012, 2013, 2014 and 2015 was 40.4, 37.2, 25.2 and 13.4 months respectively. ” (line 252)

**Supplementary table 1: Follow-up time of the 3810 patients who started ART in Zimbabwe between 2012 – 2015.**

| **Year** | **Observations** | **Observed follow-up time in months** | |
| --- | --- | --- | --- |
|  |  | **Minimum** | **Maximum** |
| 2012 | 289 | 0.09 | 40.4 |
| 2013 | 1476 | 0.1 | 37.2 |
| 2014 | 1850 | 0.1 | 25.2 |
| 2015 | 195 | 0.1 | 13.4 |

3. **Statistical analysis: Line 203-5 “****Time to attrition was calculated as the time between the date of ART initiation and the date of outcome or last patient clinic visit. Those active on ART and transferred out were censored on the date of data abstraction and the date they were transferred out.” I don’t understand the second sentence. How can you be censored to two dates?**

Those who were active on ART were censored on the date of data abstraction. Those who were transferred out were censored on the date that they were transferred to another clinic.

We revised the sentence as follows;

“Time to attrition was calculated as the time between the date of ART initiation and the date of outcome or last patient clinic visit. Those active on ART and transferred out were censored on the date of data abstraction and the date they were transferred out, respectively.” (lines 207-210)

4. **As you don’t have information on the time of censoring you could think of using the AFT-Model (Accelerated-Failure-Time-Modell) instead of the Cox-Model. In the AFT-Model you don’t need a date but a period in which an event/censoring occurs. Did you check for the proportional hazards assumption of the Cox-Model?**

Many thanks for the suggestion. Since this is a follow-up paper to an earlier evaluation, we decided to use the same methods as in the previous study, which allowed us to interpret similarities and differences between the findings of both studies. The assumptions of the Cox-Model have been checked and now included in the paper. The methods section has been expanded as follows*;*

“The proportional hazards assumption was assessed using log-log plots and Kaplan-Meier versus Cox predicted plots and results of these analyses suggested that the proportional hazards assumption holds” (lines 206-208)

5. **I suggest to look into subgroups of patients conditioning on the time having been in the ART-program: e.g. the retention in those patients already having been in ART for 6 months/for 12 months. Maybe the risk of attritions is time dependent and decreases over time.**

We assessed whether the hazard of attrition was constant overtime using cumulative hazard (Figure 1, shown here below, but not in the revised manuscript) and smooth hazard estimate curves. Overall the hazard seems to be reasonably constant over time. Moreover, we did an additional analysis to assess 12-month attrition, excluding those who started in 2015 as most of these patients had a maximal follow-up time that was lower than 12 months. This analysis is explained under query 8, and shown as figure 3. The results from this analysis are coherent with results shown in the multivariable regression model: patients who started in more recent years were more at risk of attrition.

**Figure 1: Nelson – Aalen cumulative hazard estimate**

6. **Fig 3: In the method section you describe an hierarchical variable selection for a multivariable model. I would have preferred a more knowledge-based model building – if at all. Could you please mention all the variables selected? I guess the reader is supposed to find out in Fig 3 – but I find bivariate p-values <0.1 and no effect estimate of this variable in the multivariable section – for example in the case of marital status. (what does the N.S. – non significant mean in this situation then?). Actually I don’t think you need the multivariable analysis as you want to describe risk groups for higher attrition. The risk groups are well described already in the bivariable analysis. A multivariable model can be used as a prediction model – but this is not what you wanted or what is wanted in dealing with ART-patients, I guess. In the results section you discuss the single risk factors in comparison to the former analysis – you don’t refer to the a prediction.**

We wish to identify risk factors, or predictors. We did not wish to develop a prediction rule.

Significant risk factors in a bivariate model may be wrong if they are the result of confounding from other variable. E.g. if height is associated with an outcome, “gender” would appear in a bivariate analysis, even when there is absolutely no gender association, simply because gender is strongly associated with height.

The explanatory variables included in the bivariable and multivariable regression are of clinical relevance for HIV outcomes. We did not wish to estimate the correlation between one primary variable of interest and attrition and did not upfront identify potential confounders to be included in the model, regardless of their association with outcome. Instead, we did wish to identify predictors of attrition using an exploratory approach. We preferred a multivariable model, to explain attrition with the variables available.

Following your recommendation, we revised our approach. Instead of only including variables into our multivariable model based on statistical significance at 0.1; sex and level of care were included in the model despite p – value > 0.1 due their clinical and biological relevance in HIV treatment outcomes (knowledge-based component).

We added some explanation in the legend of the table, to enhance clarity and simplify the final multivariable model. When “NS” (Not significant) it means that the variable was not significantly associated with attrition at level 0.05 and was not included in the final model.

We have expanded and revised the methods as follows;

“To identify independent risk factors for attrition, first variables were selected into the multivariable model based using a stepwise forward approach (p-value < 0.1). Moreover, sex and level of care were included based on their clinical and biological relevance in HIV retention. Second, stepwise backward elimination was used until all remaining variables were significantly associated with attrition (p-value < 0.05)”(lines 212-216)

7. **Fig 2 and Fig 3. Please refrain from reporting the p-values besides your main effect (please see the paper ‘Scientists rise up against statistical significance’ in Nature 2019** [**https://www.nature.com/articles/d41586-019-00857-9**](https://www.nature.com/articles/d41586-019-00857-9)**)**

Thank you for this comment and for providing the reference. We have removed some p-values. The p-values for multivariable analysis were maintained as they played a crucial role in variable selection of the multivariate model. We want to stress that the predictors we found are relevant for our setting, but due to the exploratory nature of the study are far from guaranteed to be associated with retention in different settings: the p-values only reflect this observed association.

8. **Fig 3:- In the sub group 2012/2013 there is much more PT compared to the patients included in 2014/2015 yielding in lower attrition rate/PT. I think this is because your sampling until 12/2015 limits the potential observation time differentially and therefore fewer persons can be present at 12 months or later in the later cohort. How can somebody in the later sub- cohort be observed for 36 months? The observation time for somebody starting in 2015 is <12 months.**

We developed supplementary table 1. Indeed, patients did not have the same maximum follow-up time. Therefore we did an additional analysis, for which we limited follow-up time to 12 months to assess the correlation between year of ART initiation and 12-month attrition, excluding those who started ART in 2015. We have expanded the methods section as follows:

“In an additional analysis we estimated the correlation between year of ART initiation and attrition until 12 months of ART. Patients who started ART in 2015 were excluded since their maximal follow-up time was too short.” (lines 216-219)

For this bivariate analysis, with follow-up time restricted to 12 months, and with those who started ART in 2015 excluded, findings are shown in the revised results section:

“We assessed the 12 months retention by year of ART initiation with those who started ART in 2015 excluded. The retention decreased during the period. Those who started ART in 2013 and 2014 as compared to those who started ART in 2012 were 2.18 and 3.78 times at risk of attrition respectively (Figure 3).” (lines 254-256)

Figure 3 has been revised and shows the 12-month survival curves, by year of ART initiation.

9. **You state that the difference in attrition rate between patients starting in the two periods is because of the changing criteria for ART-therapy regarding the cell count. in the introduction and conclusion you write that the change in this criterium happened in 2013. According to Fig 1 most of the patients in the cohort 2012/2013 should have had the same conditions for inclusion into the program than during 2014/2015.**

The World Health Organization announced the policy change in 2013. In Zimbabwe, the policy change was announced in 2013 but nationwide implementation of the recommendation started around 2014. Initially there was the adaptation process, sensitization and capacity building, pilot implementation and later gradually implementation within the provinces This explains why complete roll out of the guidelines happened in the first quarter of 2014. Obviously there could have been some overlap in the implementation of different guidelines but we expect that the impact on our findings minimal.

10. **Maybe this paper could be of interest for you as well: Alhaj M et al. Retention on antiretroviral therapy during Universal Test and Treat implementation in Zomba district, Malawi: a retrospective cohort study. J Int AIDS Soc 2019 Feb;22(2):e25239**

Many thanks for sharing this reference. We appreciate the methods used and have adopted our own manuscript. We added the analysis described above using 12-month follow-up time, and more explicitly state in the methods the component using knowledge based model building. In terms of the results there were similarities in risk of attrition among young adults. However, the findings contradict in terms of risk of attrition for those starting under UTT and pregnant women. In our case UTT was partially implemented.

We refer to the manuscript using reference number 52.

11. **Please check the syntax of the sentences in lines 283 and 390**

Many thanks for noticing the errors. The sentences have been corrected,

“Attrition was mainly explained by LTFU” (line 284)

“We also found that patients who started ART in 2013 or later were at risk of attrition as compared to those who started in 2012” (lines 403-404 )

12. **Loss-To-Follow-Up not abbreviated correctly all the time – please check!!**

Many thanks. We use lost to follow-up (LTFU) throughout the revised version.
